# A Manual of Military Surgery

**Published:** 1861-06

**Authors:** 


A MANUAL OF MILITARY SURGERY, OR HINTS ON THE EMERGENCIES OF FIELD, CAMP AND HOSPITAL PRACTICE.—By S. D. GROSS, M. D., Professor of Surgery in the Jefferson Medical College, of Philadelphia. J. B. Lippincott & Co. Philadelphia: 1861.

This is a little 16mo of 186 pages, originally prepared for a Magazine-article, but afterwards changed to a manual for the instruction of surgeons to the volunteer forces of the army. The subjects treated of are the Duties of Surgeons, Hospitals, Stores, Wounds, Amputations and Resections, Diseases of Camp, Military Hygiene, Examination of Recruits, and Dietetic Formulœ. The work was evidently prepared in haste, and hence is not up to the usual standard of the writings of the distinguished author; but, from its portability, and the amount of concise information it contains, will, no doubt, be favorably received by the junior members of the army medical staff.

